# P-1608. Inactivating Effect of 222-nm Far Ultraviolet-C Irradiation on Aerosolized SARS-CoV-2 and SARS-CoV-2 in Solution

**DOI:** 10.1093/ofid/ofaf695.1787

**Published:** 2026-01-11

**Authors:** Hiroki Kitagawa, Toshihito Nomura, Keitaro Omori, Norifumi Shigemoto, Hiroki Ohge

**Affiliations:** Hiroshima University Hospital, Hiroshima, Hiroshima, Japan; Hiroshima University Hospital, Hiroshima, Hiroshima, Japan; Hiroshima University Hospital, Hiroshima, Hiroshima, Japan; Hiroshima University Hospital, Hiroshima, Hiroshima, Japan; Hiroshima University Hospital, Hiroshima, Hiroshima, Japan

## Abstract

**Background:**

Severe acute respiratory syndrome coronavirus 2 (SARS-CoV-2) is transmitted by droplets, aerosols, and contact. Recent studies have demonstrated the effect of 254-nm, 265-nm, and 280-nm UVC on inactivating aerosolized SARS-CoV-2. This study aimed to investigate the in vitro efficacy of 222-nm far ultraviolet-C (UVC) light on the inactivation of airborne SARS-CoV-2 and SARS-CoV-2 in solution.

**Figure:**
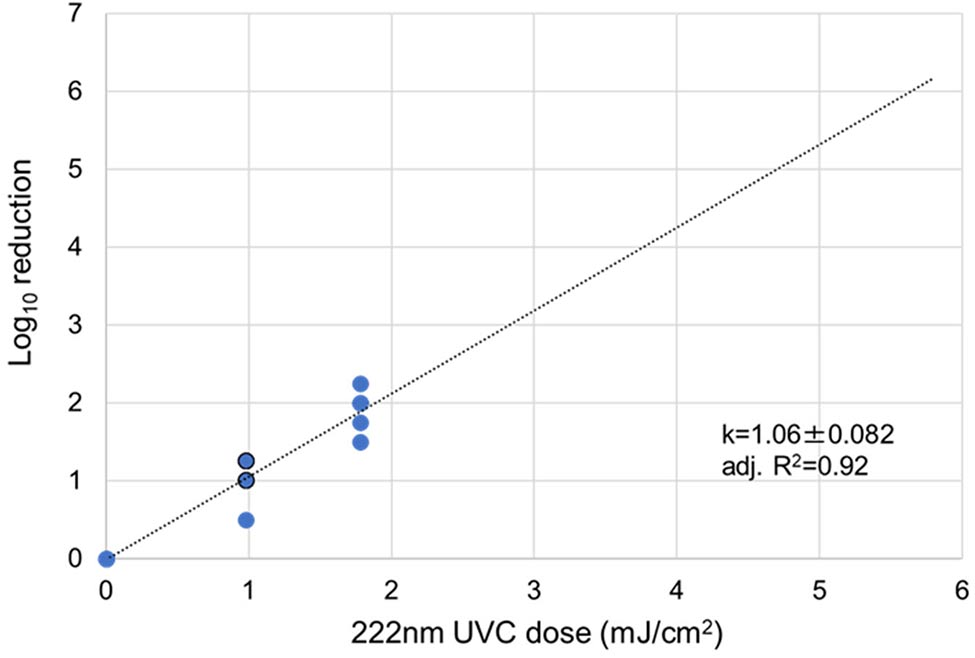
Dose response of aerosolized SARS-CoV-2 to 222-nm UVC irradiation Dashed lines represent linear regression results computed from experimental data. The mean (± standard error) pseudo-first-order inactivation rate constant (k) values (cm2/mJ) and adjusted R2 values are listed. Solid symbols with a black edge represent two samples with the same UV dose response overlapping in the plot.

**Methods:**

Virus inactivation experiments were performed using our custom-built benchtop aerosol irradiation chamber which generated, exposed, and collected aerosols containing SARS-CoV-2. The effect of 222-nm UVC on inactivation of SARS-CoV-2 in thin-film solution was also determined. The titer of SARS-CoV-2 was investigated using the 50% tissue culture infectious dose (TCID_50_). SARS-CoV-2 solution of approximately 10^8^ TCID_50_/mL in Dulbecco's modified Eagle's minimum essential medium (DMEM) without fetal calf serum was used.

**Results:**

The mean (± standard error) inactivation rate constants for virus solutions diluted 10-fold and 100-fold in PBS were 0.89 (± 0.043) cm^2^/mJ and 1.13 (± 0.057) cm^2^/mJ, respectively. The mean (± standard error) inactivation rate constant for aerosolized SARS-CoV-2 was 1.06 (± 0.082) cm^2^/mJ (Figure). The number of copies of SARS-CoV-2 RNA in the samples at each fluence were similar.

**Conclusion:**

The inactivation effect of 222-nm UVC irradiation on aerosolized SARS-CoV-2 was similar to that on SARS-CoV-2 in DMEM diluted 100-fold in PBS. Further evaluation of efficacy of 222-nm UVC irradiation in inactivating airborne SARS-CoV-2 under real-world conditions is needed.

**Disclosures:**

All Authors: No reported disclosures

